# Vancomycin-Induced Severe Neutropenia in a Patient With Osteomyelitis: A Case Report

**DOI:** 10.7759/cureus.81135

**Published:** 2025-03-25

**Authors:** Imad Majeed, Zaraq R Khan, Julie Harting, Lyndsey Armes, Forest W Arnold

**Affiliations:** 1 Infectious Diseases, University of Louisville Hospital, Louisville, USA; 2 Pharmacology, Sullivan University College of Pharmacy and Health Sciences, Louisville, USA; 3 Infectious Diseases, University of Louisville School of Medicine, Louisville, USA

**Keywords:** adverse drug reactions, neutropenia, side effects, vancomycin trough level, white blood cell

## Abstract

Vancomycin is a glycopeptide antibiotic with activity against Gram-positive bacteria, including methicillin-resistant *Staphylococcus aureus* (MRSA). We present a 52-year-old Caucasian HIV-positive male patient who initially presented to an outside hospital with altered mental status and drainage of purulence from his submental area and was started empirically on IV meropenem and IV vancomycin. He was nonadherent on antiretroviral therapy (ART) with elvitegravir/cobicistat/emtricitabine/tenofovir. His CD4 count was 1,063 cells/mm³ (64%) and undetectable viral load. A maxillofacial scan revealed findings suggestive of chronic osteomyelitis of the mandible for which surgical debridement was performed with positive cultures for methicillin-resistant *Staphylococcus epidermidis* (MRSE). He was transferred to our facility for further care on hospital day six. His intravenous vancomycin was continued, while IV meropenem was stopped. At our facility, his baseline serum creatinine (SCr) was reported at 0.51 mg/dL, and his white blood cell count (WBC) and absolute neutrophil count (ANC) were 9,900 cells/mm³ and 6,000 cells/µL, respectively. After 13 days of treatment, notable neutropenia and nephrotoxicity occurred. On day 14 of therapy, the WBC and ANC decreased acutely to 1,600 cells/mm³ and 900 cells/µL, respectively; the SCr increased acutely to 1.23 mg/dL; and a vancomycin trough level resulted in 34.7 mg/L. The corresponding vancomycin area under the curve (AUC) was 852 mg/L, which exceeds the recommended AUC goal range of 400-600 mg/L. The vancomycin AUC was within a goal range four days prior, and there was no evidence of nephrotoxicity or neutropenia at that time. Despite vancomycin dosage reductions and holding ART, neutropenia persisted. He was eventually switched to daptomycin, and neutropenia resolved after one week. The patient received vancomycin for 13 days before experiencing neutropenia. In this patient's case, concurrent acute kidney injury and supratherapeutic vancomycin concentrations may have contributed to neutropenia. Of note, his other medication known to cause potential neutropenia was discontinued, which included elvitegravir/cobicistat, emtricitabine, and tenofovir alafenamide. While literature demonstrates a prolonged duration of vancomycin treatment with neutropenia, his case is unique because it illustrates an association between neutropenia and vancomycin exposure. Vancomycin-induced neutropenia could be multifactorial, relating not just to duration but also to supratherapeutic vancomycin levels. This report aims to describe a unique case of vancomycin-induced neutropenia in a patient who exhibited persistent supratherapeutic drug levels in addition to a prolonged course of treatment.

## Introduction

Vancomycin, a glycopeptide antibiotic effective against Gram-positive organisms, is the primary treatment for methicillin-resistant *Staphylococcus aureus *(MRSA) infections. Its utilization is associated with numerous adverse effects, including nephrotoxicity, ototoxicity, hypersensitivity reactions, neutropenia, and infusion reactions [1]. Furthermore, adverse effects have been reported less frequently. Nephrotoxicity is the predominant adverse effect, contingent upon total drug exposure, as evidenced by an area under the curve exceeding 600 mg*hr/mL. A specific risk factor for vancomycin-induced neutropenia is an extended treatment duration rather than being dose-dependent (based on total daily dosage or supratherapeutic serum levels) [2].

The available data suggest that the incidence of severe neutropenia or agranulocytosis associated with non-chemotherapy drug exposure ranges from approximately 1.6-15.4 cases per million people annually. The medications most frequently linked to these conditions include dipyrone, diclofenac, ticlopidine, calcium dobesilate, spironolactone, antithyroid agents (e.g., propylthiouracil), carbamazepine, sulfamethoxazole-trimethoprim, β-lactam antibiotics, clozapine, levamisole, and vancomycin [3].

Vancomycin-induced neutropenia is commonly characterized by an absolute neutrophil count (ANC) of fewer than 1,000 cells/µL [4]. The incidence of neutropenia resulting from vancomycin is anticipated to range from 2% to 12%, as determined by retrospective studies [5]. Neutropenia typically manifests after a minimum of 12 days of vancomycin therapy, although it has been observed as early as seven days post-treatment commencement [6,7]. Re-exposure to vancomycin may result in a more rapid onset of neutropenia for patients with a prior history of vancomycin-induced neutropenia [6]. In most instances, neutrophils spontaneously recover following the cessation of the causative substance. Neutropenia generally resolves within 48-72 hours, although the duration may be prolonged in individuals undergoing hemodialysis [7]. In specific instances of severe drug-induced agranulocytosis, granulocyte colony-stimulating factor (GCSF) filgrastim has been employed as an adjunctive therapy, as it may expedite the recovery of neutrophils [8].

This report aims to elucidate a rare instance of vancomycin-induced neutropenia in a patient who exhibited sustained supratherapeutic drug concentrations and renal impairment in the context of an extended duration of therapy.

This article was presented at the Research Louisville conference as a poster on September 16, 2024.

## Case presentation

**Figure 1:**
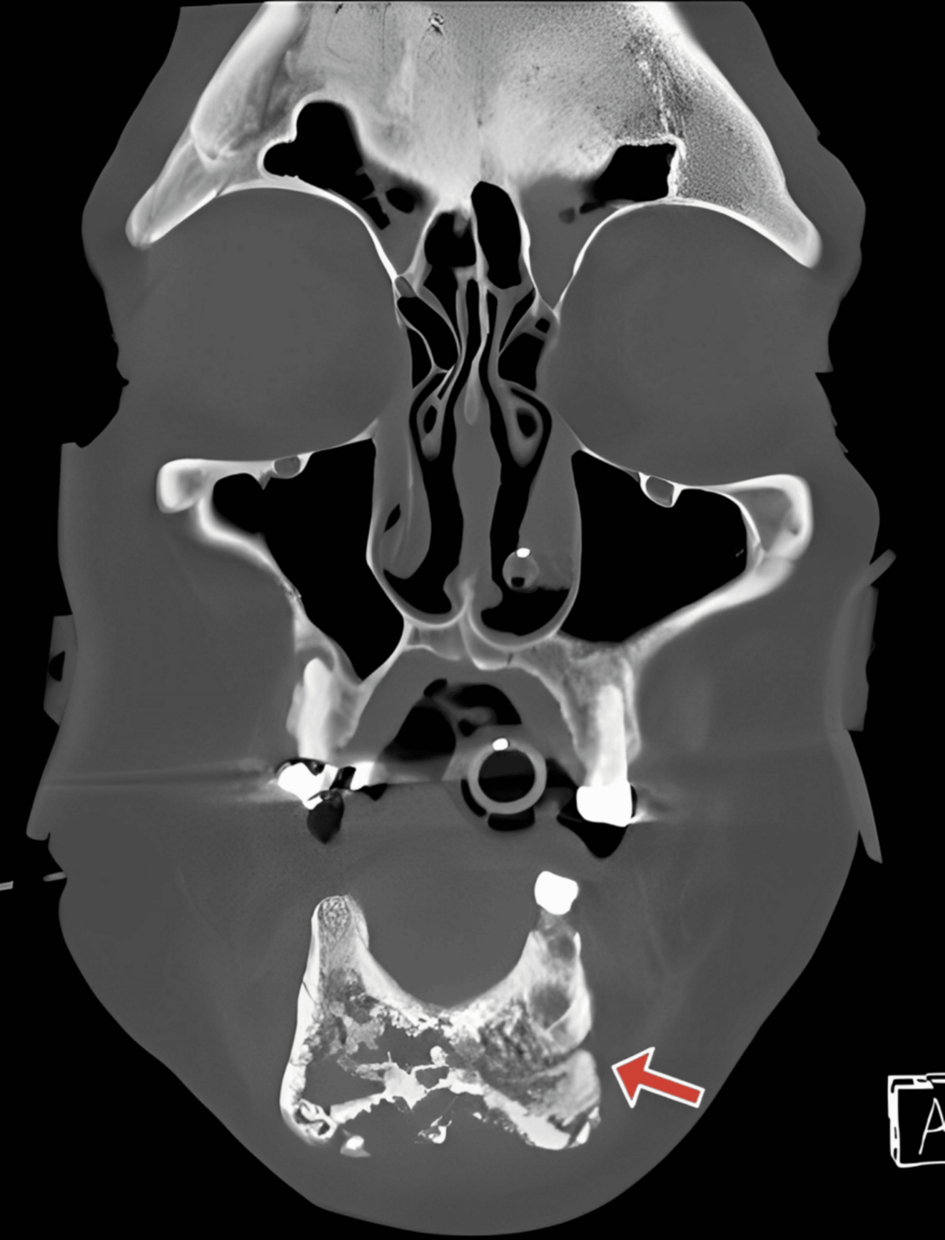
This computed tomography (CT) scan presents a coronal view of the maxillofacial region, highlighting the mandibular bone exhibiting a moth-damaged appearance (indicated by the red arrow).

A 52-year-old Caucasian male patient, with a past medical history of alcohol use disorder and HIV, was initially taken to an outside hospital facility by his ex-girlfriend after being discovered unconscious in his apartment for an unknown period. On arriving at the facility, sepsis was identified as defined by systemic inflammatory response syndrome (SIRS) of 2/4: heart rate of 110 bpm and temperature of 95° Fahrenheit. His blood pressure was 80/55 mm Hg. In the emergency department, he received a 30 mL/kg IV fluid bolus, IV norepinephrine, IV meropenem, and IV vancomycin. Computed tomography (CT) of the head, cervical spine, abdomen/pelvis, and chest was performed, which was unremarkable. Physical examination revealed numerous ecchymosis, edema, and purulent discharge from the patient's right jaw, prompting an order for CT of the neck soft tissue without IV contrast, which revealed a moth-eaten irregular appearance to the anterior mandible with cortical breakthrough, most likely caused by osteomyelitis and accompanying pathological fracture (Figure 1).

A complete blood count showed a white blood cell count (WBC) of 9,900 cells/mm³ (4,000-10,000 cells/µL), hemoglobin of 8.7 g/dL (13.5-17.5 g/dL), and platelets of 300,000 cells/µL (150,000-450,000/µL). His HIV RNA viral load was undetectable, and his CD4 count was 1,063 cells/mm³ (500-1,200 cells/mm³) (63.6%). A basic metabolic panel showed serum creatinine (SCr) of 0.51 mg/dL (0.7-1.3 mg/dL) and blood urea nitrogen (BUN) of 15 mg/dL (6-20 mg/dL). Two blood cultures were drawn, both of which resulted negative after five days. A submental wound swab was sent for culture, which grew methicillin-resistant *Staphylococcus epidermidis* (MRSE) and viridans group streptococci.

On hospital day six, the patient was transferred to our facility for intensive maxillofacial and infectious disease care. By that point, the patient had already been weaned off norepinephrine. Upon arriving at our facility, oral maxillofacial surgery was consulted, who advised initial conservative therapy because the patient was deemed a poor surgical candidate. Based on the culture results, IV meropenem was discontinued. Infectious disease was consulted, and antiretroviral therapy (ART) was resumed using the patient's home regimen of elvitegravir/cobicistat, emtricitabine, and tenofovir alafenamide.

**Figure 2:**
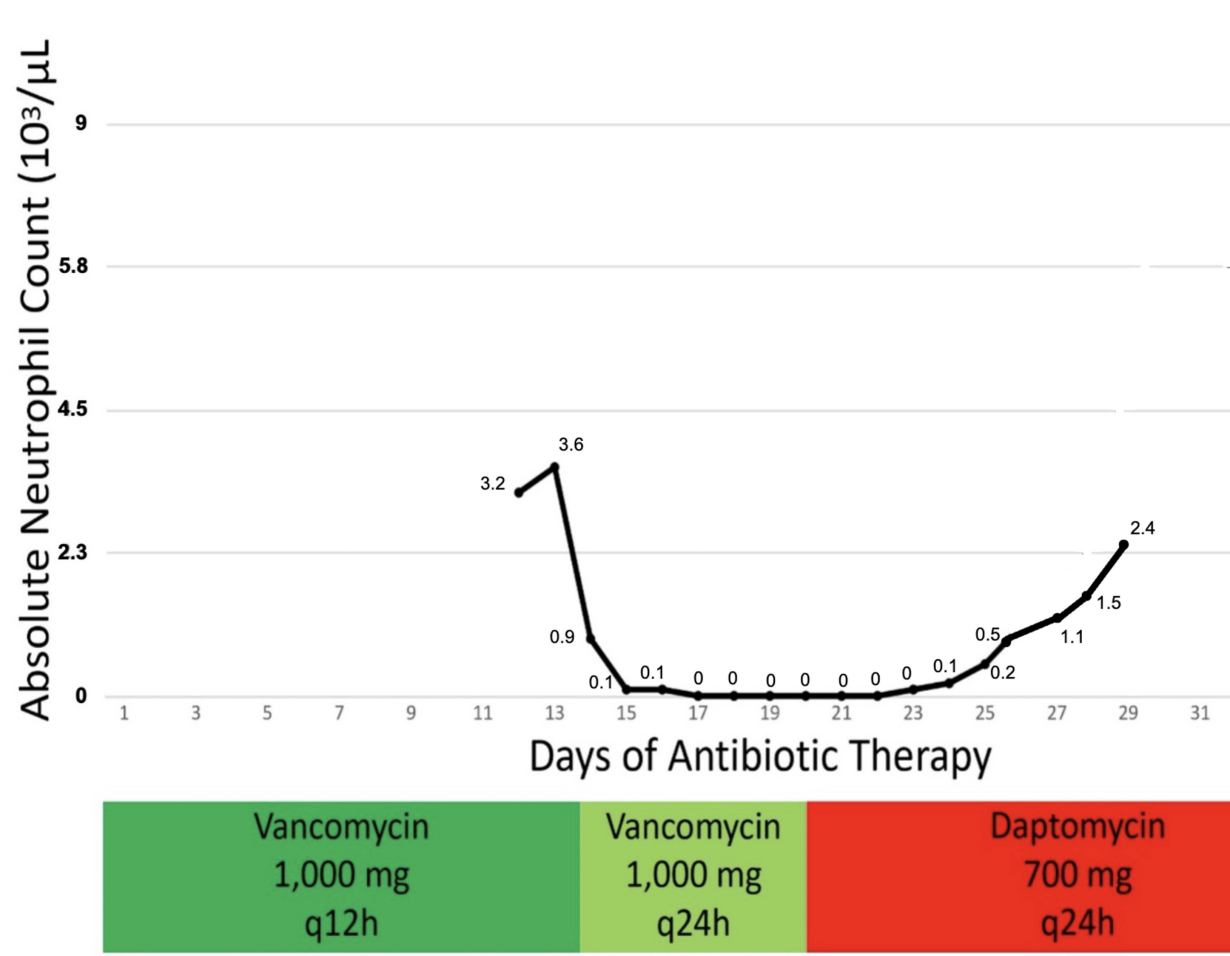
Graph revealing the absolute neutrophil count. The Y-axis during hospital course is touching the bottom line. Furthermore, the X-axis represents the duration of antibiotic therapy in the hospital course, while the colored boxes (green, light green, and red) indicate the antibiotic dosing intervals (12-hour and 24-hour doses of vancomycin and daptomycin).

**Figure 3:**
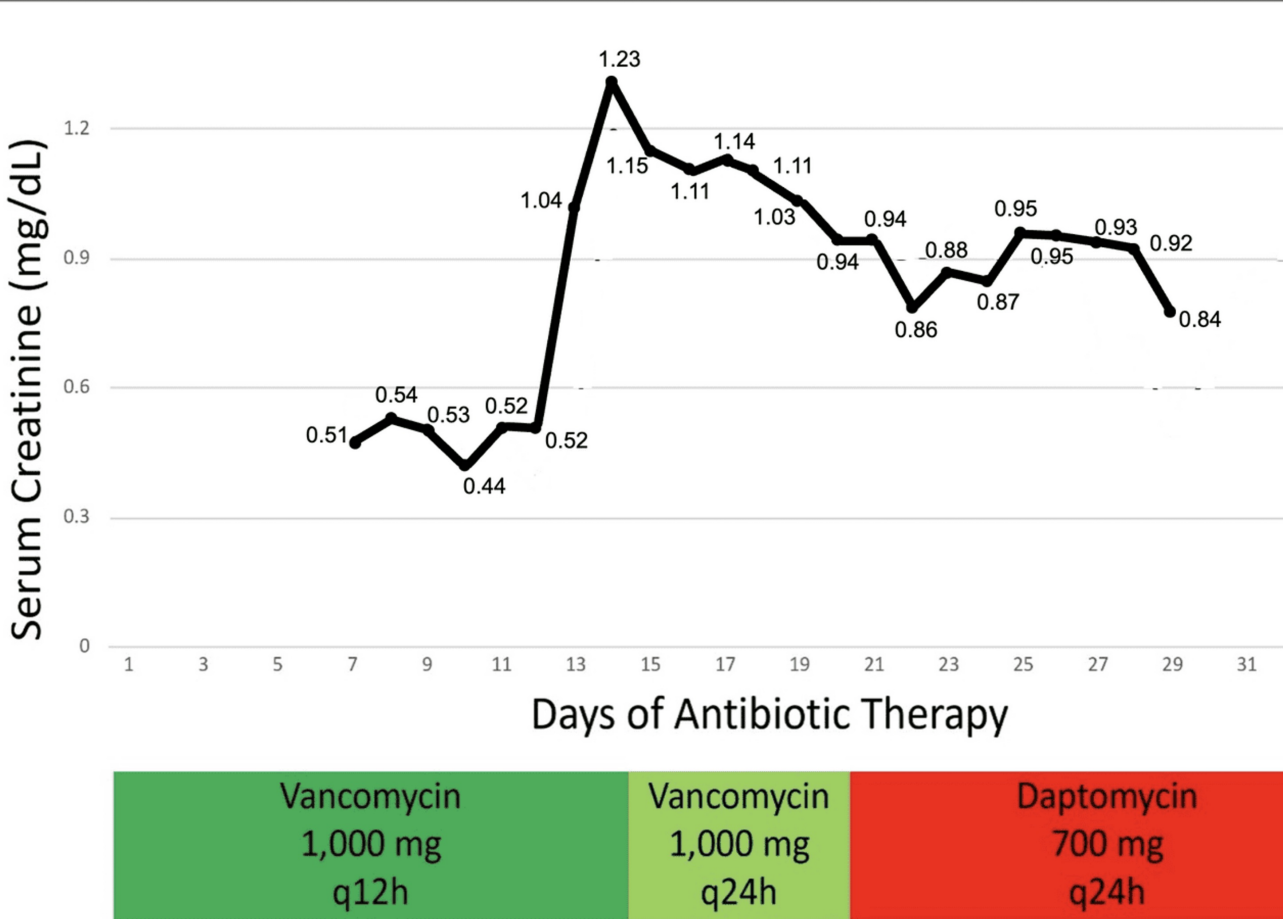
Graph revealing the serum creatinine. The Y-axis shows serum creatinine variation with the days of antibiotic therapy, with the marked high level of serum creatinine on day 14 of the antibiotic dose. Furthermore, the X-axis represents the duration of antibiotic therapy in the hospital course, while the colored boxes (green, light green, and red) indicate the antibiotic dosing intervals (12-hour and 24-hour doses of vancomycin and daptomycin).

On hospital day seven, the patient was transported to the operating room, where drainage of a submental abscess and a bone biopsy were performed, with specimens sent for culture. A bone sample was also sent to NextGen DNA sequencing. Candida *albicans *was isolated from bone culture, whereas *Fusobacterium nucleatum*, *C. albicans*, *Candida parapsilosis, *and *Candida glabrata *were identified via NextGen DNA sequencing. The patient was started on intravenous micafungin 100 mg every 24 hours, along with intravenous metronidazole 500 mg every eight hours. IV vancomycin was continued for previously isolated Gram-positive bacteria. On hospital day nine (day 14 of vancomycin therapy), WBC decreased acutely from 4,100 cells/mm³ to 1,600 cells/mm³. On the same day, the absolute neutrophil count (ANC) was reported at 900 cells/µL (1500-7,700 cells/µL), and serum creatinine increased acutely to 1.23 mg/dL (Figures 2-3).

**Figure 4:**
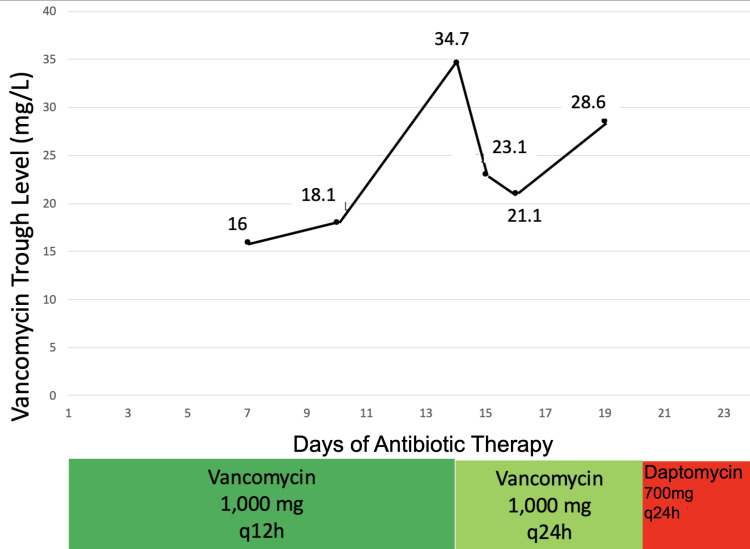
Graph illustrating the variation in vancomycin trough levels over the course of antibiotic therapy. The highest recorded level was 34.7, which occurred on day 14. Furthermore, the X-axis represents the duration of antibiotic therapy in the hospital course, while the colored boxes (green, light green, and red) indicate the antibiotic dosing intervals (12-hour and 24-hour doses of vancomycin and daptomycin).

The vancomycin serum concentration resulted in 34.7 mg/L (Figure 4).

The corresponding vancomycin AUC was 852 mg*h/L, which exceeds the recommended AUC goal range of 400-600 mg*h/L. The next vancomycin dose was held, and subsequent doses were managed by the pharmacy. Of note, the vancomycin AUC was within the goal range four days prior, and there was no evidence of nephrotoxicity or neutropenia at that time. ART was discontinued after a thorough medication review was conducted to identify potential medication-related causes of neutropenia. After several additional days of vancomycin therapy, during which serum concentrations remained supratherapeutic despite significant dose reductions, WBC remained low (1,200 cells/mm³), prompting the decision to stop vancomycin and begin daptomycin 8 mg/kg on hospital day 20 of the antibiotic course. One week after discontinuing vancomycin, WBC increased to 5,600 cells/mm³ on hospital day 29 of the antibiotic course. The patient was eventually discharged on daptomycin and metronidazole to complete his course for a total of six weeks and micafungin for at least three months (Table 1).

**Table 1:** Correlation between the days of antibiotic administration, supra-therapeutic levels of vancomycin, and absolute neutrophil count.

Days of hospital antibiotic course	Antibiotic doses and intervals	Levels and Interpretation	Serum creatinine (0.7-1.3 mg/dL)	WBC (4.5-11 k/uL)	Absolute neutrophil count (1.5-7 k/uL)
7	IV Vancomycin 1000 mg q12h	Therapeutic	0.51	9.9	6
8	IV Vancomycin 1000 mg q12h	Therapeutic	0.54	7.9	5.7
9	IV Vancomycin 1000 mg q12h	Therapeutic	0.53	6.4	4.2
10	IV Vancomycin 1000 mg q12h	Therapeutic (18.1)	0.44	5.3	3.7
11	IV Vancomycin 1000 mg q12h	Therapeutic	0.52	6.1	4.5
12	IV Vancomycin 1000 mg q12h	Probably becoming supratherapeutic	0.52	5.3	3.2
13	IV Vancomycin 1000 mg q12h	Probably supratherapeutic but level was not measured	1.04	4.1	3.6
14	IV Vancomycin 1000 mg q12h	Supratherapeutic (34.7)	1.23	1.6	0.9
15	IV Vancomycin 1000 mg q12h	Supratherapeutic (23.1)	1.15	0.9	0.1
16	IV Vancomycin 1000 mg q12h	Supratherapeutic (21.1)	1.11	1.4	0.1
17	IV Vancomycin 1000 mg q12h	Supratherapeutic	1.14	1.5	0
18	IV Vancomycin 1000 mg q12h	Supratherapeutic	1.11	1.3	0
19	IV vancomycin 750 mg q12h	Supratherapeutic (28.6)	1.03	1.1	0
20	IV Daptomycin 750 mg q24h	Still with vancomycin in system since it takes time to fully excrete	0.94	1.2	0
21	IV Daptomycin 750 mg q24h	Levels not required	0.94	1.7	0
22	IV Daptomycin 750 mg q24h	Levels not required	0.86	1.7	0
23	IV Daptomycin 750 mg q24h	Levels not required	0.88	0.8	0
24	IV Daptomycin 750 mg q24h	Levels not required	0.87	1.6	0.1
25	IV Daptomycin 750 mg q24h	Levels not required	0.95	2	0.2
26	IV Daptomycin 750 mg q24h	Levels not required	0.95	1.7	0.5
27	IV Daptomycin 750 mg q24h	Levels not required	0.93	3.2	1.1
28	IV Daptomycin 750 mg q24h	Levels not required	0.92	3.9	1.5
29	IV Daptomycin 750 mg q24h	Levels not required	0.84	5.6	2.4

## Discussion

Neutrophils are essential to the innate immune system, and drug-induced neutropenia elevates the risk of severe infections, septicemia, and septic shock in patients [3].

The lifespan of neutrophils is variable and can suddenly present as neutropenic fever. Neutropenia is often characterized by an ANC below 1,500 cells/µL; however, severe neutropenia, referred to as agranulocytosis, is defined as an ANC below 500 cells/µL [3].

The mechanism of drug-induced immune neutropenia (DIIN) is inadequately understood, and several ideas have been proposed, including a direct harmful effect [3]. Vancomycin-induced neutropenia is a diagnosis of exclusion, necessitating the elimination of other potential causes, including but not limited to malignancy, chemotherapy, and other antibiotics. Our patient was not undergoing any chemotherapy and tested negative for any malignancy, including the CAT scan of the chest, abdomen, and pelvis. We discontinued his antiretroviral medication, which comprised elvitegravir/cobicistat, emtricitabine, and tenofovir alafenamide, on the 14th day of hospitalization due to its potential as a confounder; however, his neutropenia persisted until we ceased vancomycin administration.

Reduced renal clearance is seen as a risk factor for this adverse medication reaction; nevertheless, a study of the literature indicated that most patients had sufficient renal function [6]. Literature and prior case reports indicate the necessity of vigilant monitoring for patients administered vancomycin for durations beyond seven days [2].

The literature research also indicates that vancomycin-induced reversible neutropenia is linked to an extended treatment duration; however, we did not identify any studies, to our knowledge, that have coupled neutropenia with supratherapeutic vancomycin doses, as reported in our instance. In our case, the patient faced a considerable risk of vancomycin-induced neutropenia due to extended vancomycin exposure necessary for osteomyelitis, elevated serum concentrations, and nephrotoxicity [9].

In our patient, on the day neutropenia manifested, his creatinine level doubled from baseline, coinciding with elevated therapeutic levels of vancomycin. His sudden emergence of aberrant markers on the same day created two potential interpretations. The nephrotoxicity may have resulted from supratherapeutic vancomycin levels (the most prevalent cause), or, conversely, nephrotoxicity may have led to elevated vancomycin levels. Our reasoning favored the initial hypothesis as we modified the vancomycin dosage; nonetheless, it failed to attain the optimal level but did result in normal creatinine levels. The etiology of the reversible neutropenia remained ambiguous, whether attributable to the supratherapeutic vancomycin or the extended treatment duration or both, as multiple vancomycin adjustments stabilized the creatinine level. However, the neutropenia persisted in the context of high supratherapeutic levels until the antibiotic was changed, leading to recovery of the neutropenia. Literature indicates that neutropenia frequently arises after a minimum of 12 days of vancomycin therapy, although it has been noted as early as seven days post-treatment commencement [5-7]. In our case, neutropenia was noted on the 14th day of vancomycin treatment. Spontaneous recovery of neutrophils may necessitate a duration of 1-22 days with a median of six days following the discontinuation of antibiotics [10]. In our patient, his neutrophil count normalized on the eighth day following the cessation of vancomycin. The author was unable to obtain the bone and battery cultures pathology slides, which would have enhanced the study’s accuracy. This is one of the limitations of this study.

## Conclusions

This case illustrates a patient who experienced neutropenia during vancomycin therapy on the same day that acute kidney injury and supratherapeutic vancomycin concentrations were noted. We believe that this is not merely a coincidence and is a case of vancomycin-induced neutropenia since the neutrophil count recovered shortly after discontinuation of therapy. The cause of neutropenia may be multifactorial, associated with both the duration of treatment and supratherapeutic vancomycin serum concentrations. While this was a rare and unexpected adverse effect, it is important for clinicians to be attentive to vancomycin dosage and duration, as well as other modifiable risk factors (e.g., other medications) for neutropenia to enhance medication management.
